# Supplementary material for: Comparative Analysis of Gene Expression in Fibroblastic Foci in Patients with Idiopathic Pulmonary Fibrosis and Pulmonary Sarcoidosis
Source: Cells. 2022 Feb 14;11(4):664. doi: 10.3390/cells11040664 (PMC8870272; doi:10.3390/cells11040664)
Supplement: Supplementary file 1 [file cells-11-00664-s001.zip › Supplementary files/Figure S1.pdf]

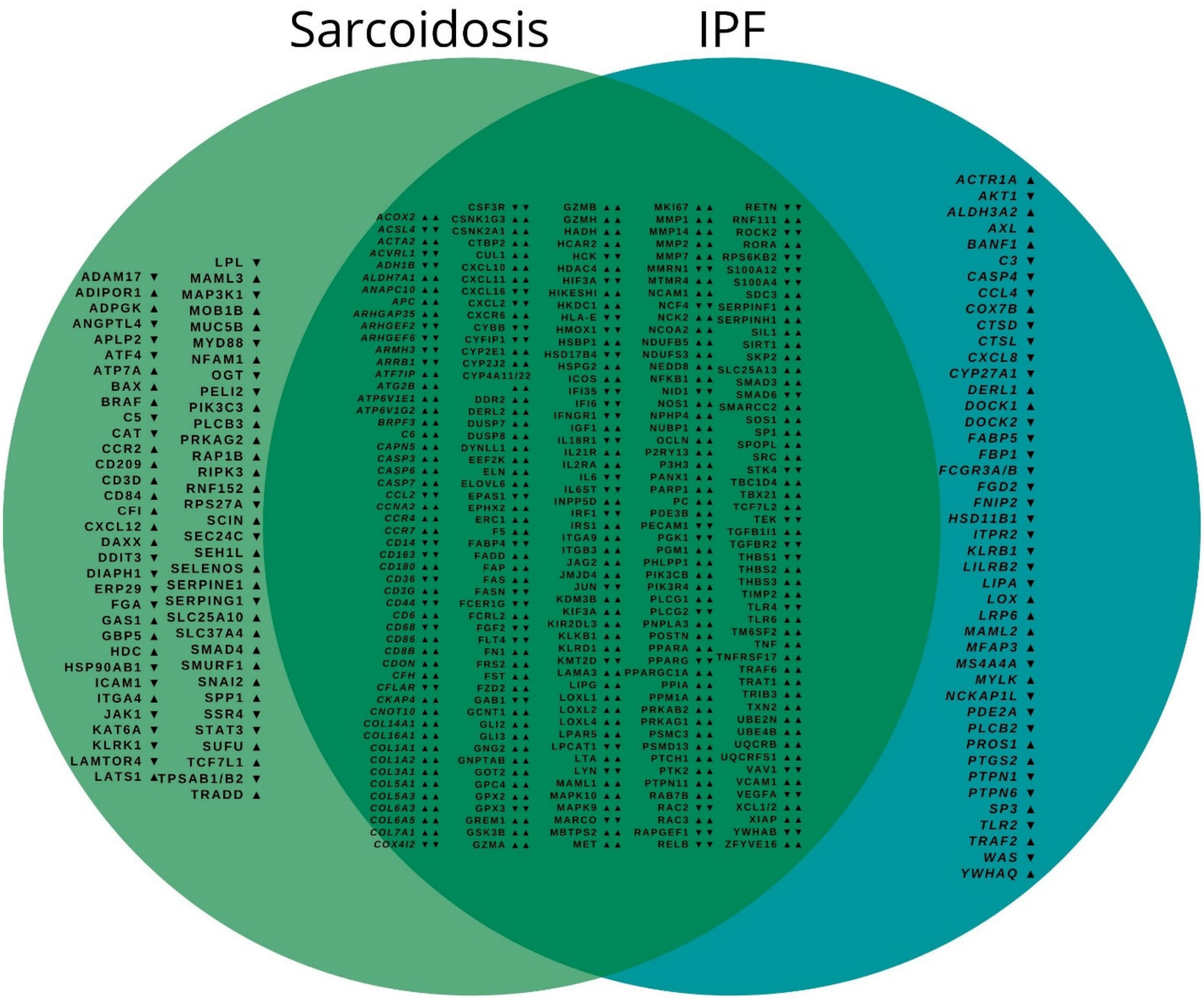

Figure S1. Venn diagram of differentially regulated genes. Arrows indicate significantly increased (arrow up) or signif-icantly decreased (arrow down) expression of the respective genes compared to controls in fibroblastic foci from both disease entities compared to healthy control lungs. False discovery rate (fdr) values <0.05 were considered as statistically significant. In the overlapping area (middle), left and right arrow indicate the expression in sarcoidosis and IPF, respectively. IPF, idiopathic pulmonary fibrosis.
