# Supplementary material for: Comparative Analysis of Gene Expression in Fibroblastic Foci in Patients with Idiopathic Pulmonary Fibrosis and Pulmonary Sarcoidosis
Source: Cells. 2022 Feb 14;11(4):664. doi: 10.3390/cells11040664 (PMC8870272; doi:10.3390/cells11040664)
Supplement: Supplementary file 1 [file cells-11-00664-s001.zip › Supplementary files/Table S1.pdf]

Table S1. Clinical information of the study group.

Patient #	Sex	Age (years)	Diagnosis
1	Female	57	UIP/IPF
2	Male	61	UIP/IPF
3	Male	43	UIP/IPF
4	Male	56	UIP/IPF
5	Male	64	UIP/IPF
6	Male	63	UIP/IPF
**Median (IQR25-75)**		**59 (56.25-62.5)**	
7	Female	65	Sarcoidosis
8	Female	32	Sarcoidosis
9	Male	55	Sarcoidosis
10	Male	53	Sarcoidosis
11	Male	58	Sarcoidosis
12	Female	57	Sarcoidosis
**Median (IQR25-75)**		**56 (53.5-57.8)**	
13-33	N/A*	N/A*	Healthy controls

UIP, usual interstitial pneumonia; IPF, idiopathic pulmonary fibrosis; IQR, interquartile range; N/A, not available; *information not publicly accessible due to data protection regulations and respecting the anonymity of donors
