# Supplementary material for: Comparative Analysis of Gene Expression in Fibroblastic Foci in Patients with Idiopathic Pulmonary Fibrosis and Pulmonary Sarcoidosis
Source: Cells. 2022 Feb 14;11(4):664. doi: 10.3390/cells11040664 (PMC8870272; doi:10.3390/cells11040664)
Supplement: Supplementary file 1 [file cells-11-00664-s001.zip › Supplementary files/Table S2.pdf]

Table S2. Target antibodies used for immunohistochemistry

Target of antibody	Product name	Host species	Pretreatment	Dilution	Manufacturer
**FAP**	Anti-Fibroblast activation protein, alpha antibody (ab53066)	Rabbit	Tris-EDTA buffer	1:100	Abcam
**MMP7**	Recombinant Anti-MMP7 antibody [EPR17888-101] (ab205525)	Rabbit	Tris-EDTA buffer	1:500	Abcam
**VCAM1**	Recombinant Anti-VCAM1 antibody [EPR5047] (ab134047)	Rabbit	Tris-EDTA buffer	1:500	Abcam
**CD86**	Anti-CD86 antibody [EP1158Y] - N-terminal (ab53004)	Rabbit	Tris-EDTA buffer	1:200	Abcam
**Collagen V**	Anti-Collagen V antibody (ab7046)	Rabbit	Tris-EDTA buffer	1:100	Abcam
**Collagen VI**	Recombinant Anti-Collagen VI antibody [EPR17072] (ab182744)	Rabbit	Tris-EDTA buffer	1:200	Abcam
**Ki-67**	Ki-67 Recombinant Monoclonal Antibody (SP6)	Rabbit	Tris-EDTA buffer	1:100	Thermo Scientific

FAP, fibroblast activation protein alpha; MMP7, matrix metalloproteinase 7; VCAM1, vascular cell adhesion molecule 1; CD86, cluster of differentiation 86; Ki-67, marker of proliferation Ki-67;
