# Supplementary figures and images for: Comparative Analysis of Gene Expression in Fibroblastic Foci in Patients with Idiopathic Pulmonary Fibrosis and Pulmonary Sarcoidosis
Source: Cells. 2022 Feb 14;11(4):664. doi: 10.3390/cells11040664 (PMC8870272; doi:10.3390/cells11040664)

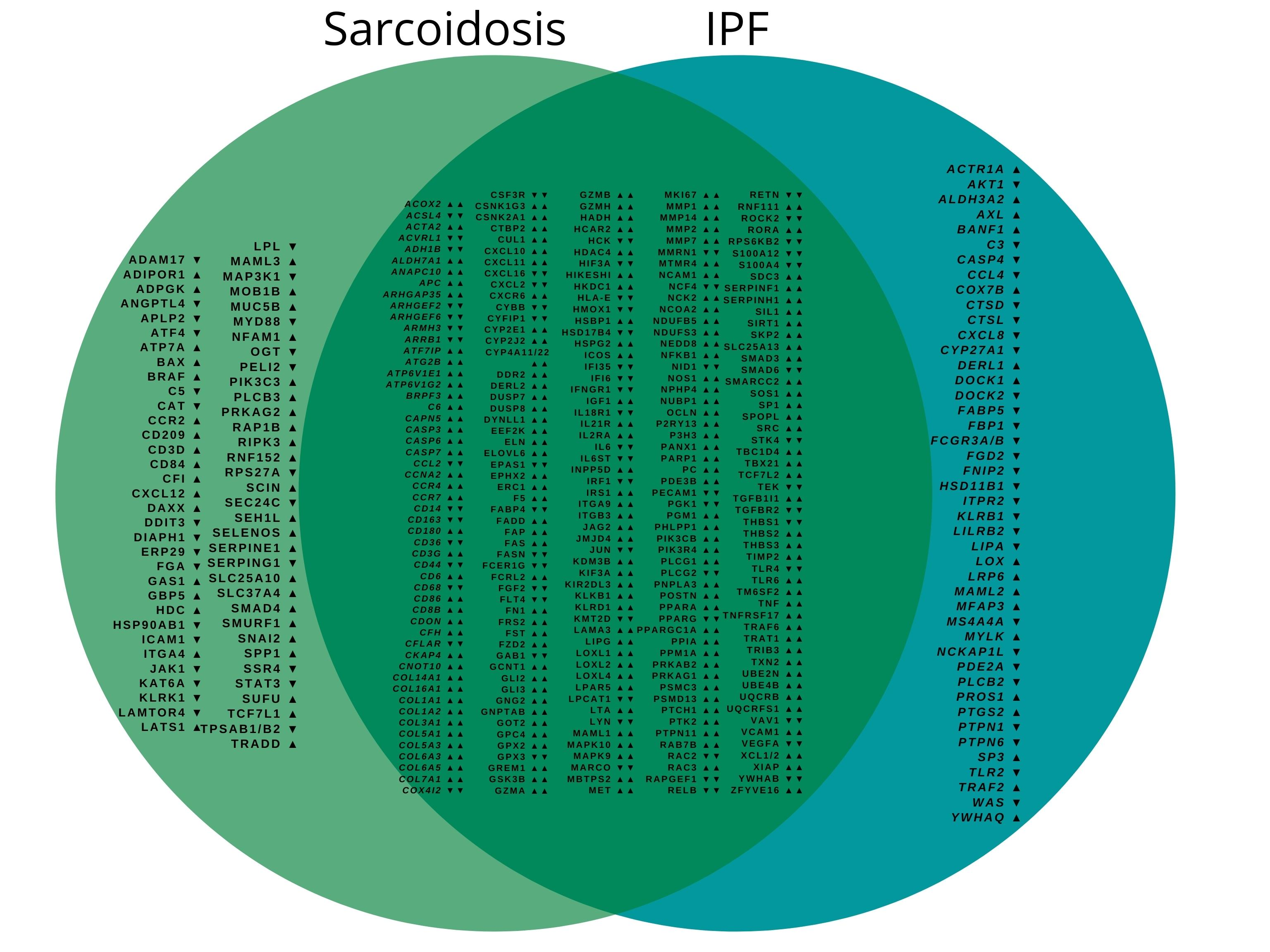

Supplement: Supplementary file 1 [file cells-11-00664-s001.zip › Supplementary files/Figure S1.tif]
